# Active bacterial communities of pig fecal microbiota transplantation suspension prepared and preserved under different conditions

**DOI:** 10.1186/s13568-019-0787-4

**Published:** 2019-05-10

**Authors:** Chunhui Lin, Jiajia Wan, Yang Lu, He Zhang, Xue Chen, Yong Su, Weiyun Zhu

**Affiliations:** 1 Laboratory of Gastrointestinal Microbiology, Jiangsu Key Laboratory of Gastrointestinal Nutrition and Animal Health, College of Animal Science and Technology, Nanjing Agricultural University, Nanjing, 210095 China; 2 Institute of Animal Husbandry and Veterinary Science, Shanghai Academy of Agricultural Sciences, Shanghai, 201106 China

**Keywords:** Active bacteria, Anaerobe, Cryopreservation, Fecal microbiota transplantation

## Abstract

Although fecal microbiota transplantation (FMT) has become a research hotspot, studies on comparison of the active fecal bacteria suspension under different preparation conditions are limited. This study investigated the abundances of active bacterial community in pig FMT suspension that produced under different oxygen concentrations or cryopreservation conditions. Fecal samples from a Landrace × Yorkshire sow were used to prepare fecal bacteria suspension under the anaerobic (AN group) and aerobic conditions (AE group), respectively. And then half of the anaerobic fecal bacteria suspension was cryopreservation in − 80 °C (AN-CR group) for 1 week. The microbial RNA in the fecal bacteria suspension was extracted before and after cryopreservation, and reverse transcribed into cDNA. MiSeq sequencing 16S rRNA gene of bacterial cDNA showed that the bacterial diversity in the AN group was significantly higher than that in the AE group. Comparing with the sows’ fecal sample, the relative abundances of *Lactobacillus johnsonii*, *Lactobacillus coleohominis* and *Parabacteroides merdae* in AN, AE and AN-CR groups were reduced. The short-term cryopreservation had low impact on the structure of the active bacterial community in the fecal bacterial suspension. These results suggest that fecal bacteria suspension can be better prepared under strict anaerobic condition, and that fecal bacteria suspension can be cryopreserved in − 80 °C for a short time.

## Introduction

Fecal microbiota transplantation (FMT) is transplantation of fecal microbiota suspension from a healthy donor to a recipient (Khajah 2017). Recently, FMT has attracted the attention of many researchers, because of its potential ability of gut microbiota restoration (Smits et al. 2016). FMT has become a very successful treatment strategy for recurrent *Clostridium difficile* infection (Debast et al. 2014), and has an obvious therapeutic effect on ulcerative colitis (Bennet and Brinkman 1989; Borody et al. 2003) and inflammatory bowel disease (Anderson et al. 2012). However, some previous studies found that FMT could induce clinical remission, but its effectiveness was not significant (Scaldaferri et al. 2016; Hu et al. 2017). This means that the effect of FMT may be variable, and the efficacy of FMT may be related to the activity of fecal bacteria. The preparation method and the transplantation procedure of fecal microbiota suspension are controversial, there is no relevant standard procedure for FMT in the current.

The efficacy of FMT is affected by a number of factors, the first step of FMT is to mix feces into fecal suspension. The gastrointestinal tract contains a large variety of microorganisms, and most of which are strictly anaerobic bacteria (like *Bacteroides*, *Prevotella* and *Ruminococcus*). To improve the efficiency of transferring active donor bacteria and reduce the time of exposuring to oxygen during the process of FMT, the pretreatment is carried out under anaerobic conditions. However, it is difficult to be strictly anaerobic during the process of collecting feces and separating microorganisms. Loose anaerobic condition could change the microbial composition of the feces and further affect the efficiency of FMT. Therefore, it is needed for us to compare the active bacteria in FMT suspension prepared under different oxygen concentrations.

Additionally, make sure the donor’s feces are fresh, feces are treated soon enough. Some researchers believe that the time from collecting feces to transplanting into the recipient’s intestinal tract preferably within 6 h (Smits et al. 2013). The isolated microorganism samples need to be cryopreserved at − 80 °C when they can’t be transplanted immediately. It is generally believed that most bacteria can restore growth and reproductive capacities when cellular environment rising to a suitable temperature range from freezing state. But the process of cryopreservation and thawing may affect the activity of some important microorganisms. A previous study showed that the clinical efficacy of cryopreserved fecal suspension in the treatment of Crohn’s disease tended to decrease compared with fresh fecal liquid (Cui et al. 2015), which suggests that cryopreservation may alter the activity of some bacteria in fecal bacteria suspension. In spite of this, there is no standard for cryopreserved preservation of donor fecal bacteria suspension.

It is generally believed that active bacteria will transcribe more rRNA for ribosome biosynthesis than inactive bacteria (Prosser 2002), so the rRNA molecules can be used as an indicator of microbial activity (Anderson and Parkin 2007). Previous studies reported that reverse transcription PCR amplification based on 16S rRNA could explore active bacteria and archaea, and some researches reflected the active part in total microbiota according to the RNA-derived sequences representing the content of bacterial ribosomes (Anderson and Parkin 2007; Baldrian et al. 2012). Therefore, this study aimed to compare the active bacterial communities of FMT suspension prepared and preserved under different conditions by high-throughput sequencing technology. The findings may provide references for the standard procedure of FMT and strategy for intestinal microbiota recovery and health improvement.

## Materials and methods

### Feces collection

Fresh feces of a healthy 110-day-gestation sow (Landrace × Yorkshire) from a commercial farm in Jiangsu province were collected into valve bag filled with CO_2_ or valve bag without CO_2_), respectively. The sow manure was transported to the laboratory by ice box and was processed within 2 h after collection. The FMT suspension prepared under anaerobic conditions or aerobic conditions are defined as the anaerobic group (AN) or aerobic group (AE), respectively. In addition, fresh sow feces were collected and stored in a centrifugal tube at − 80 °C for the analysis of active fecal microflora (sows’ fecal group, SF).

### Preparation of fecal bacteria suspension

The preparation of donor fecal bacteria suspension was adapted from a previous method (Hamilton et al. 2012). Two hundred and fifty milliliter sterile NaCl solution (0.9%) with CO_2_ was added into 50 g feces of the AN group, and the same volume of NaCl solution (0.9%) without CO_2_ was added to the AE group. The outcomes were filtered with sterilized gauze to remove the large particles, and then the filtrate was divided into centrifugal tubes. It was noted that the anaerobic group was continuously inlet into CO_2_ during filtration operation. The turbid liquid obtained by filtration was centrifuged at 2000*g*/min for 5 min, and the supernatant was collected as the bacteria suspension. In addition, part of bacteria suspension of the anaerobic group was separated, added with 10% sterile glycerin, and stored in − 80 °C for a week (anaerobic-cryopreserved group; AN-CR).

### RNA extraction and reverse transcription

RNA was isolated from active bacteria in feces and FMT suspension of three groups with Trizol reagent (Invitrogen, CA) according to the instruction. The concentration of extracted RNA was determined by using a Nano-Drop 1000 spectrophotometer (Thermo Scientific Inc., Wilmington, DE, USA). The RNA was then diluted to 500 ng/mL, and was reverse transcribed to cDNA using Primer Script TM RT Reagent Kit (Takara, Japan) following the instruction. The cDNA was preserved at − 20 °C for further sequencing of 16S rRNA gene of active bacteria.

### PCR Amplification, Illumina MiSeq sequencing and bioinformatics analysis

**Fig. 1:**
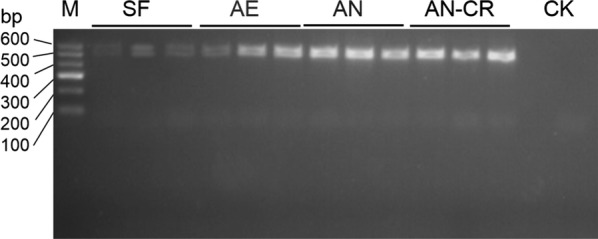
PCR products of the V3-V4 region of bacterial 16S rRNA gene with primers 341F/806R. *SF* sows’ fecal group, *AE* aerobic group, *AN* anaerobic group, *AE-CR*Anaerobic-cryopreserved group, *CK* control check

Primers 341F (5′-CCTAYGGGRBGCASCAG-3′) and 806R (5′-GGACTACNNGGGTATCTAAT-3′) were selected to amplify the V3–V4 region of bacterial 16S rRNA gene by polymerase chain reaction (PCR) (Hjelmsø et al. 2014). The PCR reaction were operated in 20 μL reaction system consisting of 2 μL dNTPs (2.5 mM), 4.0 μL 5× buffer, 0.8 μL forward and reverse primer (5 μM), 0.4 μL FastPfu polymerase, 1 μL template cDNA and 11 μL sterile water. The amplification conditions were as follows: pre-denaturation at 95 °C for 2 min; and then there were 25 cycles: denaturation at 95 °C for 30 s, anneal at 55 °C for 30 s, extension at 72 °C for 30 s; the final extension at 72 °C for 5 min (Sun et al. 2015). The PCR products of the same sample were mixed and detected by agarose gel electrophoresis (Fig. 1), and then the AxyPrepDNA gel Recovery Kit (Axygen Biosciences, Union City, CA, US) was used to recover PCR products. After eluted by Tris–HCl, the PCR products were sequenced on Illumina MiSeq platform.

In order to strictly control the quality of effective sequences, QIIME (1.17) was used to filter the raw data by deleting sequences containing barcode tags, sequences with length less than 150 bp, sequences with base ambiguity or mismatch more than two, and sequences with more than six repeats of a single base (Caporaso et al. 2010). UPARSE (version 7.1, http://drive5.com/uparse/) was used for operational taxonomic units (OTU) clustering analysis of the selected sequences, and sequence whose similarity reached 97% were classified into a class (Edgar 2013). In order to compare the differences of bacterial diversity among different samples, all samples were randomly sampled to a uniform amount of data according to minimum number of sequences in all samples. Finally, the sequence number of all samples was 28173. RDP classifier (Release 11.1, http://rdp.cme.msu.edu/) Bayesian algorithm was used to analyze the community composition of each OTU at each classification level such as domain, kingdom, phylum, class, order, family, genus and species (Cole et al. 2008). Mothur software was used to do rarefaction analysis on the OTU level of bacteria, the abundance-based coverage estimator (Ace), the bias-corrected Chao richness estimator, and the Shannon and Simpson diversity indices were also calculated. The Bray–Curtis similarity clustering analysis of the abundance of OTUs was used to perform a principal coordinates analysis (PCoA) (Bray and Curtis 1957). The raw sequencing reads were submitted to Sequencing Read Archive (SRA) database under the accession id: SRP169828.

### Data statistics and analysis

Statistical software Statistical Package for the Social Sciences (SPSS v20) was used for data statistics. The one-way ANOVA test was performed to analyze the differences in bacterial communities of fecal bacteria suspension among groups. Three replicates were used for each group (n = 3). Significant differences were declared when *P *< 0.05.

## Results

### Bacterial sequencing and bacterial diversity analysis

**Fig. 2:**
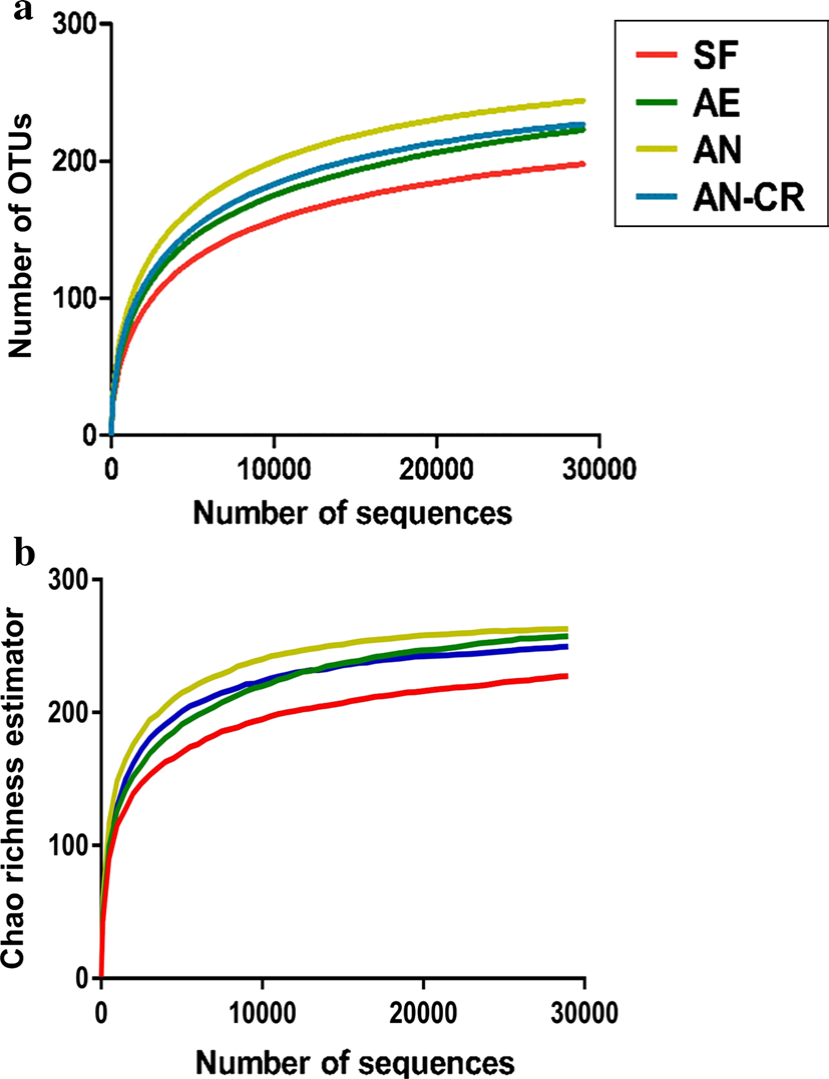
Rarefaction curves plotting the number of phylotypes (**a**) and Chao richness estimator (**b**) in the 16Sr RNA gene libraries by the number of sequences from active microbiota in all groups. *SF* sows’ fecal group, *AE* aerobic group, *AN* anaerobic group, *AE-CR* anaerobic-cryopreserved group

A total of 421,263 sequences were of bacterial origin with length greater than 250 bp, and the average sequence length was 420.5 bp. As shown in Fig. 2, with the increase of the number of sequences, the dilution curves gradually tended to approach the saturation plateau, which indicates that the sequencing depth of all the samples in this study is sufficient to reflect the composition and diversity of the microflora in each sample.

The bacterial richness estimator (Ace and Chao) of fecal bacteria suspension in different treatment conditions had no significant change (*P* > 0.05). However, bacterial Shannon diversity indices of the AN group were significantly higher than that in SF and AE groups (*P* < 0.05), bacterial Simpson diversity indices in AN and AN-CR groups were significantly lower than that in SF and AE groups (*P* < 0.05) (Table 1).

**Table 1:** Diversity and richness estimation of the active microbiota in all groups

Item	SF	AE	AN	AN-CR	SEM	*P* value
Ace	231.45	257.84	265.45	250.12	5.02	0.173
Chao	235.42	258.37	265.84	251.13	5.93	0.432
Shannon	2.45^c^	2.56^bc^	3.10^a^	2.88^ab^	0.09	0.011
Simpson	0.25^a^	0.24^a^	0.13^b^	0.16^b^	0.02	0.020
Coverage	0.999	0.999	0.999	0.999	0.00	0.333

Data are expressed as mean and standard error of means (SEM), n = 3. Means within the same row with different superscripts are significantly different from one another

*SF* sows’ fecal group, *AE* aerobic group, *AN* anaerobic group, *AN-CR* anaerobic-cryopreserved group

### Effects of different preparation and preservation conditions on the structure of active bacteria in fecal bacteria suspension

**Fig. 3:**
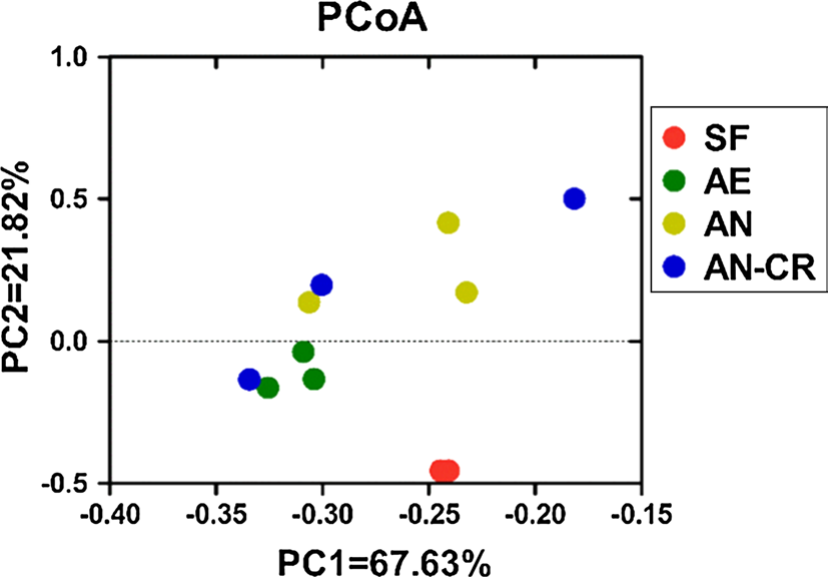
Principal coordinate analysis (PCoA) of active bacterial communities in all groups. *SF* sows’ fecal group, *AE* aerobic group, *AN* anaerobic group, *AE-CR* anaerobic-cryopreserved group

PCoA of bacterial communities at the OTU level (Fig. 3) showed that active bacterial community in the SF group was differed from microbiota in the maternal fecal bacteria suspension. Distinct bacterial communities were observed between the AE and AN groups, while no statistical significance of the spatial separation was observed between AN and AN-CR groups in PCoA plots.

**Fig. 4:**
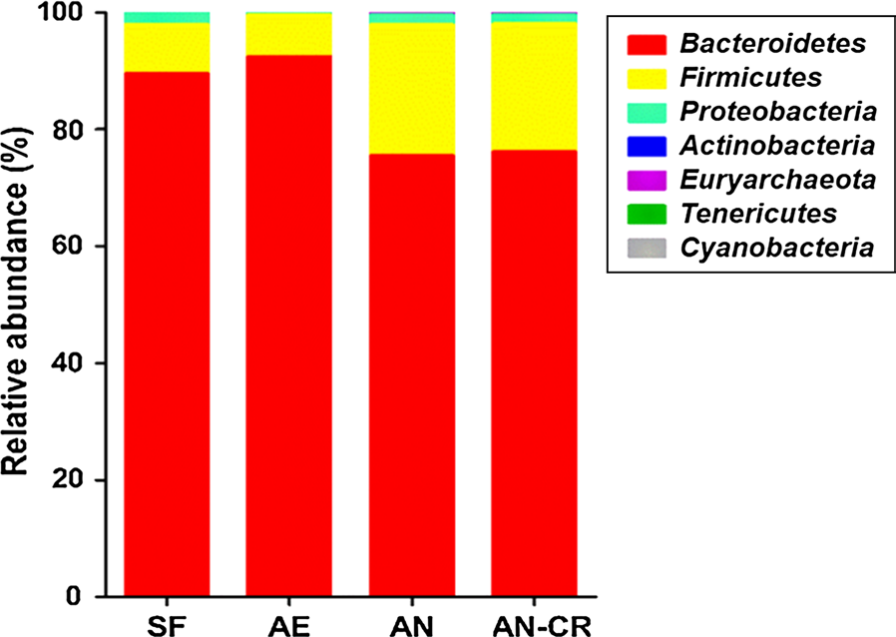
The average relative abundance of active microbial phyla in all groups. *SF* sows’ fecal group, *AE* aerobic group, *AN* anaerobic group, *AE-CR* anaerobic-cryopreserved group

At the phylum level (Fig. 4), the dominant phylum of the fecal bacteria suspension was *Bacteroidetes*, followed by *Firmicutes*, totally accounted for over 95%, but the abundance of the two dominant phyla among groups had no significant differences (*P* > 0.05). The relative abundance of *Actinobacteria* in the SF group was significantly higher than that in FMT suspension of the other three groups (*P* < 0.05). Besides, the abundance of active *Cyanobacteria* tended to increase (*P* < 0.10) under the anaerobic condition.

**Fig. 5:**
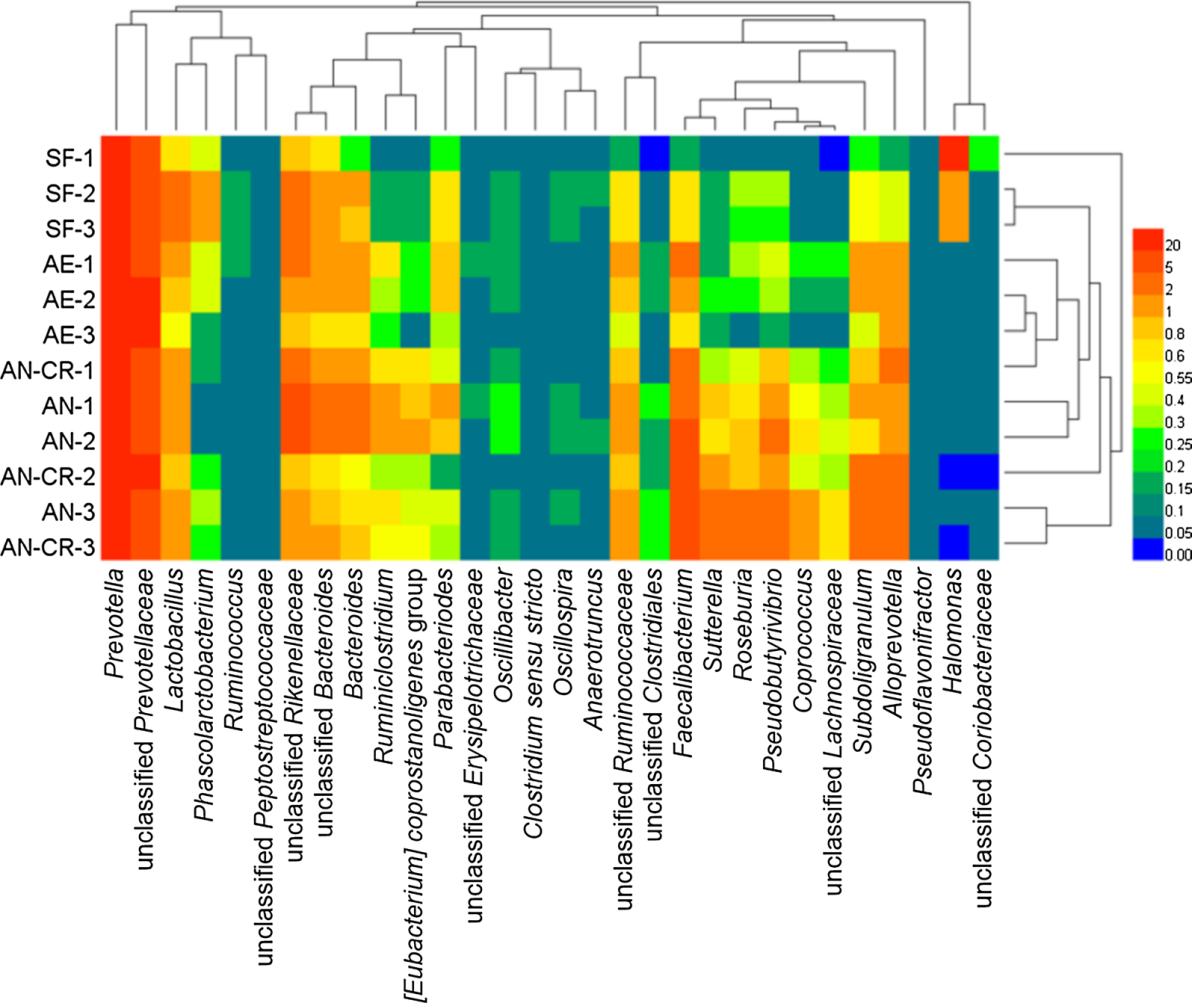
Heat map of genera in the relative abundances of active bacterial communities in all groups. *SF* sows’ fecal group, *AE* aerobic group, *AN* anaerobic group, *AE-CR* anaerobic-cryopreserved group

At the genus level (Table 2), the predominant genera were *Prevotella* and unclassified *Prevotellaceae* in all groups. Compared with the SF group, the relative abundances of *Halomonas*, unclassified *Rikenellaceae*, *Alloprevotella*, [*Ruminococcus*] *gauvreauii* group, *Blautia*, *Anaerotruncus*, unclassified *Gastranaerophilales*, *Escherichia*-*Shigella*, *Candidatus Captivus*, *Pelagibacterium*, and *Howardella* significantly decreased in FMT suspension of the other three groups (*P* < 0.05). The relative abundances of unclassified *Bacteroidales*, *Sutterella*, *Ruminiclostridium*, unclassified *Lachnospiraceae*, unclassified *Thermoplasmatales Incertae Sedis*, *Enterobacter*, *Pseudoflavonifractor*, *Aquabacterium*, *Asteroleplasma*, [*Eubacterium*] *ventriosum* group, and *Tyzzerella* in the AN group were higher than those in the AE and SF groups (*P* < 0.05). Meantime, compared with the AN group, the relative abundances of *Ruminiclostridium*, unclassified *Lachnospiraceae*, *Enterobacter*, *Campylobacter*, [*Eubacterium*] *ventriosum* group, and *Tyzzerella* in the AN-CR group decreased significantly (*P* < 0.05), whereas the relative abundances of *Prevotella* and unclassified *Prevotellaceae* in the AE group increased significantly (*P* < 0.05). The cluster analysis based on heat map demonstrated a higher similarity of the samples within group than that among groups except the AN-CR group (Fig. 5).

**Table 2:** Relative abundances of active microbial genera that were significant different among all groups

Item	SF	AE	AN	AN-CR	SEM	*P* value
*Prevotella*	73.91^a^	66.64^a^	49.07^b^	51.20^b^	3.65	0.016
Unclassified *Prevotellaceae*	10.08^c^	19.98^a^	14.76^bc^	17.89^ab^	1.24	0.010
*Halomonas*	2.95^a^	1.03^b^	1.41^b^	1.20^b^	0.24	0.003
Unclassified *Rikenellaceae*	1.65^a^	0.01^b^	0.01^b^	0.00^b^	0.20	0.000
*Alloprevotella*	1.56^a^	0.35^b^	0.15^b^	0.20^b^	0.14	0.000
Unclassified *Bacteroidales*	0.71^b^	0.79^b^	1.60^a^	1.12^ab^	0.32	0.046
*Sutterella*	0.44^c^	1.32^bc^	1.98^ab^	2.93^a^	0.24	0.023
*Ruminiclostridium*	0.17^b^	0.40^b^	0.89^a^	0.48^b^	0.01	0.010
Unclassified *Lachnospiraceae*	0.11^ab^	0.05^c^	0.12^a^	0.07^bc^	0.10	0.015
Unclassified *Thermoplasmatales Incertae Sedis*	0.10^b^	0.19^b^	0.79^a^	0.48^ab^	0.01	0.035
[*Ruminococcus*] *gauvreauii* group	0.07^a^	0.01^b^	0.01^b^	0.00^b^	0.02	0.000
*Enterobacter*	0.03^ab^	0.02^b^	0.06^a^	0.02^b^	0.01	0.042
*Campylobacter*	0.03^b^	0.09^a^	0.09^a^	0.05^b^	0.01	0.011
*Blautia*	0.04^a^	0.02^b^	0.02^b^	0.01^b^	0.03	0.016
*Pseudoflavonifractor*	0.02^b^	0.05^b^	0.13^a^	0.08^ab^	0.00	0.035
*Anaerotruncus*	0.03^a^	0.00^b^	0.00^b^	0.00^b^	0.01	0.003
*Aquabacterium*	0.01^b^	0.04^b^	0.09^a^	0.06^ab^	0.00	0.010
Unclassified *Gastranaerophilales*	0.01^a^	0.00^b^	0.00^b^	0.00^b^	0.10	0.014
*Escherichia*-*Shigella*	0.02^a^	0.00^b^	0.00^b^	0.00^b^	0.00	0.001
*Candidatus captivus*	0.01^a^	0.00^b^	0.00^b^	0.00^b^	0.00	0.048
*Pelagibacterium*	0.01^a^	0.00^b^	0.00^b^	0.00^b^	0.00	0.000
*Howardella*	0.01^a^	0.00^b^	0.00^b^	0.00^b^	0.00	0.000
*Asteroleplasma*	0.00^b^	0.01^b^	0.06^a^	0.03^ab^	0.01	0.019
[*Eubacterium*] *ventriosum* group	0.00^b^	0.00^b^	0.04^a^	0.02^b^	0.01	0.025
*Tyzzerella*	0.01^b^	0.00^b^	0.02^a^	0.01^b^	0.00	0.041

Data are expressed as mean and standard error of means (SEM), n = 3. Means within the same row with different superscripts are significantly different from one another

*SF* sows’ fecal group, *AE* aerobic group, *AN* anaerobic group, *AN-CR* anaerobic-cryopreserved group

As presented in Table 3, 67 OTU were significantly affected in the relative abundances during the preparation of maternal fecal bacteria suspension. As compared to the SF group, three maternal fecal bacteria suspension groups showed lower abundances of OTU137 (*s*_*Lactobacillus johnsonii*), OTU87 (*s*_*Lactobacillus coleohominis*), OTU43 (*o*_*Clostridiales*), OTU118 (*g*_*Aquabacterium*), OTU234 (*g*_*Helicobacter*), OTU244 (*s*_*Parabacteroides merdae*), and OTU69 (*g*_*Ruminiclostridium*) (*P* < 0.05). Compared with the AN group, the AE group showed higher abundances of OTU137 (*s*_*L. johnsonii*), OTU87 (*s*_*L. coleohominis*), OTU43 (*o*_*Clostridiales*), OTU164 (*f*_*Peptostreptococcaceae*), and OTU143 (*f*_*Prevotellaceae*) (*P* < 0.05), and lower abundances of OTU202 (*g*_*Alloprevotella*), OTU171 (*f*_*Ruminococcaceae*), OTU88 (*f*_*Peptococcaceae*), OTU285 (*g*_*Alistipes*), and OTU210 (*g*_*Terrisporobacter*) (*P* < 0.05). After 7 days of cryopreservation, the maternal fecal bacteria suspension showed higher abundances of OTU137 (*s*_*L. johnsonii*), OTU92 (*g*_*Anaerofilum*), OTU84 (*g*_[*Eubacterium*] *coprostanoligenes* group), and OTU241 (*s*_*Bacteroides uniformis*) (*P* < 0.05), and lower abundances of OTU171 (*f*_*Ruminococcaceae*), OTU88 (*f*_*Peptococcaceae*), OTU285 (*g*_*Alistipes*), OTU210 (*g*_*Terrisporobacter*), OTU183 (*f*_*Ruminococcaceae*), and OTU242 (*s*_*Bacteroides chinchillae*) (*P* < 0.05).

**Table 3:** Relative abundances of active microbial OTUs that were significant different among all groups

Item	SF	AE	AN	AN-CR	SEM	*P* value	Annotation
OTU137	8.87^a^	6.93^b^	4.82^c^	6.24^b^	0.46	0.001	*s__Lactobacillus johnsonii*
OTU87	5.14^a^	1.43^b^	0.66^c^	0.77^c^	0.52	0.000	*s__Lactobacillus coleohominis*
OTU33	2.88^b^	6.81^a^	5.11^a^	6.55^a^	0.51	0.007	*f__Ruminococcaceae*
OTU43	2.55^a^	1.24^b^	0.61^c^	0.87^bc^	0.22	0.000	*o__Clostridiales*
OTU118	2.38^a^	0.72^b^	1.03^b^	0.89^b^	0.20	0.001	*g__Aquabacterium*
OTU164	2.02^b^	3.27^a^	1.77^b^	2.26^b^	0.20	0.004	*f__Peptostreptococcaceae*
OTU143	1.96^c^	5.10^a^	3.72^b^	4.71^ab^	0.39	0.008	*f__Prevotellaceae*
OTU234	1.76^a^	0.77^b^	0.79^b^	0.39^b^	0.16	0.002	*g__Helicobacter*
OTU244	1.64^a^	0.01^b^	0.01^b^	0.00^b^	0.20	0.000	*s__Parabacteroides merdae*
OTU69	1.56^a^	0.35^b^	0.15^b^	0.20^b^	0.17	0.000	*g__Ruminiclostridium*
OTU30	0.70^b^	1.79^a^	0.95^b^	1.09^b^	0.14	0.009	*g__*[*Eubacterium*] *ventriosum* group
OTU144	0.35^a^	0.14^b^	0.17^b^	0.17^b^	0.03	0.028	*g__Parabacteroides*
OTU48	0.39^a^	0.40^a^	0.14^b^	0.15^b^	0.04	0.002	*o__Clostridiales*
OTU92	0.30^c^	2.23^ab^	1.58^b^	2.42^a^	0.26	0.004	*g__Anaerofilum*
OTU158	0.29^c^	0.65^bc^	1.06^ab^	1.52^a^	0.16	0.018	*s__*[*Pseudomonas*] *geniculata*
OTU36	0.22^a^	0.07^b^	0.06^b^	0.05	0.02	0.003	*f__Lachnospiraceae*
OTU230	0.35^b^	1.01^a^	0.79^a^	1.00^a^	0.09	0.009	*f__Ruminococcaceae*
OTU58	0.19^a^	0.08^b^	0.03^c^	0.03^c^	0.02	0.000	*g__Allisonella*
OTU251	0.10^a^	0.02^b^	0.02^b^	0.01^b^	0.01	0.007	*o__Bacteroidales*
OTU240	0.12^a^	0.09^a^	0.01^b^	0.01^b^	0.02	0.007	*f__Prevotellaceae*
OTU6	0.11^a^	0.07^ab^	0.02^b^	0.02^b^	0.01	0.030	*g__Howardella*
OTU202	0.09^b^	0.16^b^	0.70^a^	0.42^ab^	0.09	0.036	*g__Alloprevotella*
OTU171	0.08^ab^	0.03^c^	0.10^a^	0.06^bc^	0.01	0.005	*f__Ruminococcaceae*
OTU88	0.09^b^	0.05^b^	0.14^a^	0.06^b^	0.01	0.006	*f__Peptococcaceae*
OTU275	0.08^ab^	0.09^a^	0.07^b^	0.06^b^	0.01	0.025	*f__Prevotellaceae*
OTU146	0.08^a^	0.01^b^	0.02^b^	0.03^b^	0.01	0.004	*g__Alloprevotella*
OTU170	0.07^a^	0.01^b^	0.01^b^	0.00^b^	0.01	0.000	*g__*[*Eubacterium*] *coprostanoligenes* group
OTU49	0.07^a^	0.03^b^	0.01^c^	0.01^c^	0.01	0.001	*f__Ruminococcaceae*
OTU84	0.06^c^	0.10^bc^	0.13^b^	0.20^a^	0.02	0.007	*g__*[*Eubacterium*] *coprostanoligenes* group
OTU226	0.04^a^	0.01^b^	0.01^b^	0.01^b^	0.00	0.017	*g__Oscillibacter*
OTU108	0.04^b^	0.16^a^	0.16^a^	0.24^a^	0.02	0.016	*f__Prevotellaceae*
OTU285	0.05^c^	0.08^c^	0.25^a^	0.17^b^	0.03	0.002	*g__Alistipes*
OTU210	0.05^a^	0.01^c^	0.03^b^	0.01^c^	0.01	0.000	*g__Terrisporobacter*
OTU272	0.04^ab^	0.02^b^	0.06^a^	0.05^a^	0.01	0.024	*s__Bacteroides eggerthii*
OTU291	0.03^a^	0.01^b^	0.02^ab^	0.01^b^	0.00	0.025	*g__Fusicatenibacter*
OTU183	0.03^b^	0.09^a^	0.09^a^	0.05^b^	0.01	0.011	*f__Ruminococcaceae*
OTU106	0.04^a^	0.02^b^	0.02^b^	0.01^b^	0.00	0.016	*f__Prevotellaceae*
OTU225	0.02^a^	0.00^c^	0.01^b^	0.00^c^	0.00	0.000	*g__*[*Eubacterium*] *coprostanoligenes* group
OTU242	0.02^c^	0.05^bc^	0.11^a^	0.07^b^	0.01	0.003	*s__Bacteroides chinchillae*
OTU55	0.03^a^	0.00^b^	0.00^b^	0.00^b^	0.00	0.003	*f__Ruminococcaceae*
OTU286	0.01^b^	0.02^ab^	0.08^a^	0.04^ab^	0.01	0.028	*o__Gastranaerophilales*
OTU280	0.02^b^	0.05^b^	0.13^a^	0.07^ab^	0.01	0.032	*g__Prevotella*
OTU95	0.01^c^	0.03^bc^	0.08^a^	0.06^ab^	0.01	0.010	*g__Candidatus Captivus*
OTU167	0.01^a^	0.00^b^	0.00^b^	0.00^b^	0.00	0.014	*g__Ruminiclostridium*
OTU73	0.02^a^	0.00^b^	0.00^b^	0.00^b^	0.00	0.000	*g__*[*Eubacterium*] *ruminantium* group
OTU3	0.02^c^	0.16^b^	0.32^a^	0.18^b^	0.03	0.003	*f__Ruminococcaceae*
OTU77	0.02^a^	0.00^b^	0.00^b^	0.00^b^	0.00	0.001	*f__Ruminococcaceae*
OTU181	0.01^b^	0.01^b^	0.03^a^	0.02^ab^	0.00	0.046	*g__Clostridium* sensu stricto
OTU200	0.01^b^	0.02^a^	0.01^b^	0.01^b^	0.00	0.027	*g__Alloprevotella*
OTU120	0.01^b^	0.02^b^	0.06^a^	0.05^a^	0.01	0.041	*f__Prevotellaceae*
OTU32	0.01^c^	0.10^b^	0.09^b^	0.17^a^	0.02	0.009	*f__Erysipelotrichaceae*
OTU168	0.01^c^	0.10^a^	0.05^bc^	0.09^ab^	0.01	0.015	*g__Oscillospira*
OTU150	0.01^a^	0.00^b^	0.00^b^	0.00^b^	0.00	0.001	*c__Cyanobacteria*
OTU229	0.01^a^	0.00^b^	0.00^b^	0.00^b^	0.00	0.020	*g__Oscillibacter*
OTU173	0.00^b^	0.00^b^	0.04^a^	0.02^b^	0.01	0.025	*f__Ruminococcaceae*
OTU236	0.00^b^	0.01^b^	0.06^a^	0.03^ab^	0.01	0.019	*g__Sutterella*
OTU63	0.00^c^	0.01^bc^	0.03^a^	0.02^ab^	0.00	0.031	*f__Ruminococcaceae*
OTU216	0.01^b^	0.00^b^	0.02^a^	0.01^b^	0.00	0.041	*g__Alloprevotella*
OTU257	0.00^b^	0.00^b^	0.01^a^	0.00^b^	0.00	0.022	*f__Prevotellaceae*
OTU289	0.00^b^	0.01^b^	0.03^a^	0.01^b^	0.00	0.016	*g__Blautia*
OTU62	0.00^b^	0.00^b^	0.01^a^	0.00^b^	0.00	0.023	*g__Ruminococcus*
OTU80	0.00^b^	0.02^a^	0.01^b^	0.01^b^	0.00	0.012	*g__Anaerotruncus*
OTU207	0.00^b^	0.10^a^	0.10^a^	0.12^a^	0.01	0.001	*g__Anaerotruncus*
OTU241	0.01^c^	0.07^ab^	0.04^bc^	0.11^a^	0.01	0.031	*s__Bacteroides uniformis*
OTU235	0.00^b^	0.00^b^	0.02^a^	0.01^b^	0.00	0.006	*s__Parasutterella secunda*

Data are expressed as mean and standard error of means (SEM), n = 3. Means within the same row with different superscripts are significantly different from one another

*SF* sows’ fecal group, *AE* aerobic group, *AN* anaerobic group, *AN-CR* anaerobic-cryopreserved group

## Discussion

Preparation of donor fecal bacteria suspension is one of the important steps during FMT, which determines whether the active functional flora of the donor can enter the recipient’s intestine. The gut microbiota are mainly anaerobic bacteria, and the genus *Prevotella* is major symbiotic bacteria, within which the bacterium cultured alone would die under the aerobic environment for 2 min (Ulluwishewa et al. 2015). Besides, whether the fecal bacteria suspension is fresh, it may also affect the efficacy of FMT after transplantation. This study aimed to compare the active bacteria of the fecal bacteria suspension under different preparation conditions, and provide a reference for optimizing the preparation conditions of the fecal bacteria suspension and improving the efficacy of FMT.

### Seek the feasibility of replacing feces with fecal bacteria suspension

For operating convenience, the donor feces were moved into the fecal bacteria suspension during FMT. So, here we compared differences in the active bacterial communities between feces and fecal bacteria suspension. The PCoA map demonstrated that the structure of the active microbiota between the pig feces and the fecal bacteria suspension varied, indicating the preparation of fecal bacteria suspension affected the active microbial compositions. The relative abundances of *L. johnsonii*, *L. coleohominis*, and *P. merdae* in the three fecal bacteria suspension groups were reduced in comparison with the SF group. As a probiotic strain, *L. johnsonii* could improve the growth performance, stimulate secretory immunoglobulin A production, and regulate the levels of various cytokines to improve immunity (Geier et al. 2010; Kaburagi et al. 2007). Therefore, the modification of active bacteria during the preparation of fecal bacteria suspension may impact the clinical effect of FMT.

However, previous studies reported that prepared fecal bacteria suspension had similar efficacy as fresh feces on treating *Clostridium difficile* infections in human (Satokari et al. 2015; Youngster et al. 2014). In contrast, the difference between feces and fecal suspension in active microbial composition may due to a lack of uniform standard in the preparation process (such as donor selection, dilution ratio) (such as donor selection, dilution ratio) (Hu et al. 2018). We also hypothesized that some altered bacteria were not important for maintain the homeostasis.

### Anaerobic conditions

Although the fecal bacteria suspension is for the most studies prepared under anaerobic conditions (Diao et al. 2018; Hu et al. 2017), in few reports the effect of oxygen conditions on the donor flora activity was analyzed. There are a large number of microorganisms in the feces, and most of them are strict anaerobes that fastidious about the living environment. Therefore, we speculated that preparing the fecal bacteria suspension under anaerobic conditions was beneficial to ensure the integrity of the fecal microbiota. Our results showed that the diversity of active bacteria in fecal bacterial suspension produced under anaerobic conditions was significantly higher than that in aerobic group, regardless of whether they were cryopreserved or not. And PCoA analysis showed that the microbial compositions in the AE group were differed from the AN group, indicating that preparing the fecal bacteria suspension can achieve the most active donor flora under strict anaerobic conditions, as described previously. This finding was based on the fact that the anaerobic state maintains the physiological activity of most anaerobic bacteria in the feces.

Phylum-level analysis revealed that the main phylum weren’t affected by oxygen concentration, but there were significant differences between the individual important functional microbiota in each group. At the genus level, the abundances of active *Prevotella* and unclassified *Prevotellaceae* in the fecal bacteria suspension were significantly higher under aerobic conditions. Genus *Prevotella* is a well-known anaerobic bacterium. However, the main reason for these difference is still unknown, which needs further investigation.

### Homogenization and cryopreservation

Some researchers suggested that the use of fecal weight and suspension volume to record the dose of fecal bacteria suspension didn’t accurately represent the number of flora (Zhang et al. 2012) because it was not clear about the distribution of the flora throughout the stool contents. Moreover, if the influence of bacterial sedimentation factors was not avoided during the fecal bacteria suspension preparation, it was even more difficult to obtain data sufficient to represent the feces as a whole. It may also explain the fact that the above anaerobic bacteria had a higher diversity under aerobic conditions (Hsieh et al. 2016).

The fecal bacteria suspension is usually prepared in advance, so it is necessary to investigate the effect of cryopreservation on bacterial compositions. In this study, the structure of the active flora after cryopreservation wasn’t significantly differed from the fresh anaerobic fecal flora, which is consistent with the finding described in Fouhy’s study (Fouhy et al. 2015). These results indicated that the transient cryopreservation had little impact on the activity of the flora in the fecal bacteria suspension. Previous studies found that there wasn’t significant difference in clinical efficacy compared with fresh stool samples when using standardized frozen fecal samples for *Clostridium difficile* infection (Hamilton et al. 2012; Costello et al. 2015). Therefore, cryopreserved fecal bacteria suspension could save energy and cost without loss of efficiency and safety during the process of FMT (Borody et al. 2015).

In conclusion, the present study found that the diversity of active bacteria in the fecal bacteria suspension prepared under anaerobic conditions was significantly increased compared with aerobic conditions. Whether under anaerobic or aerobic conditions, the preparation of fecal bacteria suspension changed the structure of the active bacteria in the feces of the sow. The short-term cryopreservation had low impact on the structure of the active bacteria in the fecal bacteria suspension. The method that could completely ensure the activity of fecal microbiota during the preparation of FMT suspension needs further investigation.

## Data Availability

The data supporting the conclusions of this article are all available.
